# Impacts of the SYNTAX score I, II and SYNTAX score II 2020 on left main revascularization

**DOI:** 10.1038/s41598-024-51192-7

**Published:** 2024-01-11

**Authors:** Wei-Ting Sung, Ming-Ju Chuang, Yi-Lin Tsai, Ruey-Hsing Chou, Chun-Chin Chang, Po-Hsun Huang

**Affiliations:** 1 Division of Cardiology, Department of Medicine, Taipei Veteran General Hospital, 112, No. 201, Sec. 2, Shih-Pai Road, Taipei, Taiwan; 2 School of Medicine, College of Medicine, National Yang Ming Chiao Tung University, Taipei, Taiwan; 3 Institute of Clinical Medicine, National Yang Ming Chiao Tung University, Taipei, Taiwan; 4 Department of Critical Care Medicine, Taipei Veterans General Hospital, Taipei, Taiwan

**Keywords:** Cardiology, Interventional cardiology

## Abstract

Patients with left main coronary artery disease (LMCAD) with a high SYNTAX score (SS) were excluded from randomized studies that comparing percutaneous coronary intervention (PCI) and coronary artery bypass grafting (CABG). We sought to compare PCI and CABG in the real-world practice and investigate the impact of SS I, SS II, and SS II 2020 on clinical outcomes. In total, 292 Patients with LMCAD (173 PCI, 119 CABG) treated between 2017 and 2021 were enrolled. The primary outcome was major adverse cardiovascular events (MACE), a composite of all-cause death, stroke, or myocardial infarction (MI). The mean SS I was high in both groups (PCI vs. CABG: 31.64 ± 11.45 vs. 32.62 ± 11.75, *p* = 0.660). The primary outcome occurred in 28 patients (16.2%) in the PCI group and in 19 patients (16.0%) in the CABG group without significant difference [adjusted hazard ratio, 95% CI = 0.98 (0.51–1.90), *p* = 0.97] over the follow-up period (26.9 ± 17.7 months). No significant difference was observed in all-cause mortality (11.6% vs. 11.8%, *p* = 0.93) or stroke rates (3.5% vs. 5.0%, *p* = 0.51) between groups. However, PCI was associated with higher MI (4.6% vs. 0.8%, *p* < 0.05) and revascularization rates (26% vs. 5.9%, *p* < 0.001). Prognostic value of the SS I, SS II and SS II 2020 on the primary outcome was not relevant in the PCI group. Among patients with LMCAD, PCI and CABG did not significantly differ in the composite endpoint of all-cause death, stroke, and MI. These results support the potential expansion of PCI indications in LMCAD management for whom are ineligible for CABG with complex coronary artery disease.

## Introduction

The optimal treatment for left main coronary artery disease (LMCAD), either percutaneous coronary intervention (PCI) or coronary artery bypass grafting (CABG), remains disputed. The Synergy Between PCI With Taxus and Cardiac Surgery (SYNTAX) study recommended CABG for Patients with LMCAD, particularly those with high SYNTAX scores (SS) I (≥33), citing higher adverse events rates with PCI^1^. However, 10-year follow-up results from SYNTAX study indicated no mortality differences between PCI and CABG^2^. Technological advancements in drug-eluting stents (DES), coronary devices, and interventional techniques has expanded the applicability of PCI. Landmark trials like EXCEL and PRECOMBAT showed comparable outcomes between PCI and CABG for patients with low to intermediate anatomical complexity^3,4^. Consequently, guidelines classify PCI as a class I recommendation for LMCAD with a low SS I (0–22), a Class IIA recommendation for LMCAD for an intermediate SS I (23–32), but a class IIIB recommendation for a high SS I^5,6^. However, high SS I patients with LMCAD, excluded from EXCEL and PRECOMBAT, may still treated by PCI in contemporary real-world practice^7,8^.

The SS I was developed as an angiographic scoring tool to classify anatomical complexity of coronary artery disease. Inherent limitations in the SS I have been repeatedly challenged and its applicability remains debatable^9^. After that, SS II incorporating anatomical complexity of coronary arteries and seven clinical characteristics was established to predict four-year all-cause mortality and determine the most appropriate revascularization strategy (PCI or CABG) in patients with multivessel disease or LMCAD^10^. Recently, SS II 2020 was developed using the ten-year outcomes reported in the extended SYNTAX(ES) study and has been externally validated using patient-level data from three landmark randomized trials^11^. Absolute risk deference (ARD) between PCI and CABG calculated using the SS II 2020 is being tested to support clinical decision making on revascularization^12^.

In this context, we sought to investigate clinical outcomes of patients with LMCAD undergoing PCI or CABG, including patients with high SS I and evaluate the impact of SS I, SS II, and SS II 2020.

## Methods

### Study population

In this retrospective study, patients with chronic or acute coronary syndrome who underwent PCI or CABG for de novo LMCAD (defined as ≥ 50% left main artery stenosis) between January 2017 and December 2021 at the Taipei Veterans General Hospital, a high-volume referral center in Taiwan, were included. The revascularization strategy, incorporating factors like lesion complexity, comorbidities, surgical risk, and affordability of DES, was a shared decision by physicians and patients. The Heart Team approach was preferred for intermediate or high SS patients. PCI and CABG procedures were performed as per local practice norms with intravascular imaging and stenting strategies left to operator discretion. Post-PCI patients received dual antiplatelet therapy as per guidelines^13^, with contemporary new generation DES recommended.

This study has been approved by the research ethics committee of the Taipei Veterans General Hospital (No.2022-06-005BC) and was conducted in accordance with the Declaration of Helsinki. The research ethics committee approved a request to waive of informed consent since no more than minimal risk to study subjects.

### SYNTAX score calculation

Two independent interventional cardiologists blinded to clinical outcomes (CC Chang and MJ Chuang) calculated SS I from coronary angiograms. SS II incorporated seven clinical parameters: age, creatinine clearance, left ventricular ejection fraction (LVEF), presence of LMCAD, gender, presence of chronic obstructive pulmonary disease (COPD), and presence of peripheral artery disease (PAD), alongside the SS I and predicted a mortality outcome for either PCI or CABG, leading to patient categorization into PCI, CABG, or equipoise groups. SS II 2020 incorporates two anatomical effect modifiers (SSI and the presence of three-vessel disease or LMCAD) and seven clinical prognostic factors of revascularization, including age, medically treated diabetes mellitus with or without insulin, COPD, PAD, current smoking, creatinine clearance and LVEF to predict 5-year major adverse cardiovascular events (MACE). SS II 2020 of the study population were analyzed by using the web calculator. The predicted 5-year MACE rates with PCI and 5-year MACE rates with CABG were provided. Absolute risk difference (ASD) between PCI and CABG was calculated (5-year MACE rate with PCI minus 5-year MACE rate with CABG).

### Study endpoints

The primary endpoint was MACE during follow-up, defined as a composite of all-cause mortality, stroke, or MI as per the Fourth Universal Myocardial Infarction definition^14^. MACE was analyzed hierarchically. The complete revascularization was defined as no residual stenosis ≥ 70%^15^ either after the index procedure or after staged PCI within 60 days.

### Statistical methods

Categorical variables are presented as percentages and numbers, continuous variables as mean ± standard deviation. Baseline characteristics and procedural data were compared using Student’s t-test for continuous variables and chi-square test for categorical data. Survival curves were constructed using Kaplan-Meier estimates and were compared using the log-rank test. Hazard ratios with 95% confidence intervals were reported based on the Cox regression model. A two-sided p value less than 0.05 was considered statistically significant. Data analysis used SPSS software (version 25, SPSS, Chicago, Illinois, USA).

## Results

### Patient characteristics

Between January 2017 and December 2021, 292 Patients with LMCAD were retrospectively enrolled: 173 underwent PCI, and 119 patients received CABG. Table 1 presents the baseline characteristics. Most of patents had LMCAD and three-vessel disease. The PCI group were older with more heart failure history and previous MI. However, other medical conditions and clinical presentations were similar between groups. Table 2 summarizes the SS I, SS II, and SS II 2020 between two groups. The anatomical complexity of coronary artery disease based on SS I was similar between groups (PCI vs. CABG: 31.64 ± 11.45 vs. 32.62 ± 11.75, *p* = 0.660). In the PCI group, 43.4% of patients had a high SS I, whereas 20.2% with a low score in the CABG group. Despite similar SS II-PCI scores and predicted 4-year PCI mortality rates across groups, SS II-CABG scores and predicted 4-year CABG mortality were significantly lower in the CABG group. Most SS II recommendations were equipoised between PCI and CABG. In the PCI group, only 80.9% of patients were treated following SS II recommendations, compared with 92.4% in the CABG group.

**Table 1:** Baseline characteristics.

	PCI (n = 173)	CABG (n = 119)	*P* value
Age (years)	72.00 ± 12.28	66.63 ± 9.51	<0.001
Male	139 (80.3%)	89 (74.8%)	0.259
Medical history			
Diabetes mellitus	86 (49.7%)	59 (49.6%)	0.982
Insulin use	23 (13.3%)	24 (20.2%)	0.144
Hypertension	135 (77.5%)	88 (73.9%)	0.490
Known HF	45 (26.0%)	10 (8.4%)	<0.001
COPD	13 (7.5%)	6 (5.0%)	0.400
Chronic kidney disease	63 (36.4%)	41 (34.5%)	0.731
End staged renal disease	19 (11.0%)	21 (17.6%)	0.104
Previous stroke	22 (12.7%)	13 (10.9%)	0.643
Previous myocardial infarction	30 (17.3%)	11 (9.2%)	0.050
Previous PCI	60 (34.7%)	29 (24.4%)	0.060
PAD	17 (9.8%)	16 (13.4%)	0.337
Current smoking	59 (34.1%)	54 (45.4%)	0.066
AF	17 (9.8%)	6 (5.0%)	0.136
Clinical presentation			0.506
Stable coronary artery disease	105 (60.7%)	65 (54.6%)	
Unstable angina	28 (16.2%)	17 (14.3%)	
NSTEMI	33 (19.1%)	31 (26.1%)	
STEMI	7 (4.0%)	6 (5.0%)	
LVEF (%)	51.58 ± 13.93	49.92 ± 12.99	0.305
Three-vessel disease	139 (80.3%)	99 (83.2%)	0.646
DAPT after procedure	169 (97.7%)	56 (47.1%)	<0.001
Aspirin + Clopidogrel	139 (80.3%)	56 (47.1%)	<0.001
Aspirin + Ticagrelor	22 (12.7%)	0 (0%)	
Aspirin + Prasugrel	8 (4.6%)	0 (0%)	
Anticoagulant after procedure	12 (6.9%)	8 (6.7%)	0.943
DOAC	7 (4.0%)	3(2.5%)	0.481
Warfarin	5 (2.9%)	5(4.2%)	0.545

Data are mean ± SD or n (%); CABG: coronary artery bypass grafting; COPD: chronic obstructive pulmonary disease; DAPT: dual antiplatelet therapy; DOAC: direct oral anticoagulants; HF: heart failure; NSTEMI: non-ST elevation myocardial infarction; PAD: peripheral artery disease; PCI: percutaneous coronary intervention; STEMI: ST elevation myocardial infarction.

**Table 2:** Comparison of SYNTAX scores.

	PCI (n=173)	CABG (n=119)	*P* value
Syntax score I	31.64 ± 11.45	32.62 ± 11.75	0.660
0 to 22	38 (22.0%)	24 (20.2%)	
23–32	60 (34.7%)	37 (31.1%)	
≥ 33	75 (43.4%)	58 (48.7%)	
Syntax score II PCI	41.26 ± 13.07	41.18 ± 13.86	0.962
Predicted 4-yr mortality PCI (%)	23.38 ± 22.77	24.01 ± 22.56	0.816
Syntax score II CABG	39.09 ± 12.66	35.26 ± 9.81	0.004
Predicted 4-yr mortality CABG (%)	19.97 ± 18.98	13.40 ± 10.67	<0.001
Syntax score II recommendation			0.081
PCI	23 (13.3%)	9 (7.6%)	
Equipoise	117 (67.6%)	76 (63.9%)	
CABG	33 (19.1%)	34 (28.6%)	
Syntax score II 2020			
Predicted 5-yr MACE with PCI (%)	40.19 ± 21.97	34.79 ± 18.80	0.03
Predicted 5-yr MACE with CABG (%)	35.83 ± 20.25	30.67 ± 15.75	0.02
Absolute risk difference (PCI-CABG)	4.35± 6.98	4.11± 6.94	0.779

Data are mean ± SD or n (%), CABG: coronary artery bypass grafting, MACE: major adverse cardiovascular events, PCI: percutaneous coronary intervention.

**Figure 1:**
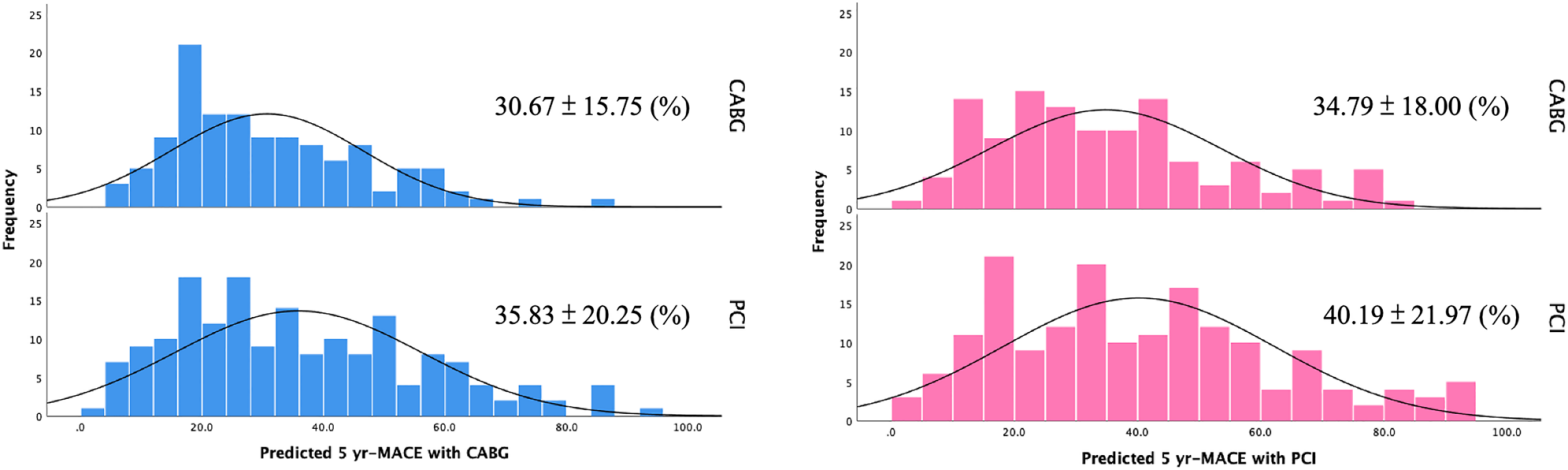
Distribution of predicted 5-year MACE rates with PCI/CABG based on SS II 2020.

The SS II 2020 predicted 5-year MACE rates with PCI and 5-year MACE rates with CABG were both significantly higher in the PCI group then the CABG group (Fig. 1 and Table 2). ARD between PCI and CABG were similar in both groups.

Supplementary Table S1 summarizes details of PCI procedures. Of PCI patients, 89.1% used intravascular imaging (either intravascular ultrasound (IVUS) or optical coherence tomography (OCT)), 70.5% received one stent for LMCAD and proximal optimization technique (POT) was performed in 87.9% of cases. Complete revascularization was achieved in 68.2% patients. We used DES in 96.5% of the patients. In the CABG group, 73.1% received arterial grafts, averaging 3.0 ± 0.8 grafts per patient.

### Major clinical outcome

**Figure 2:**
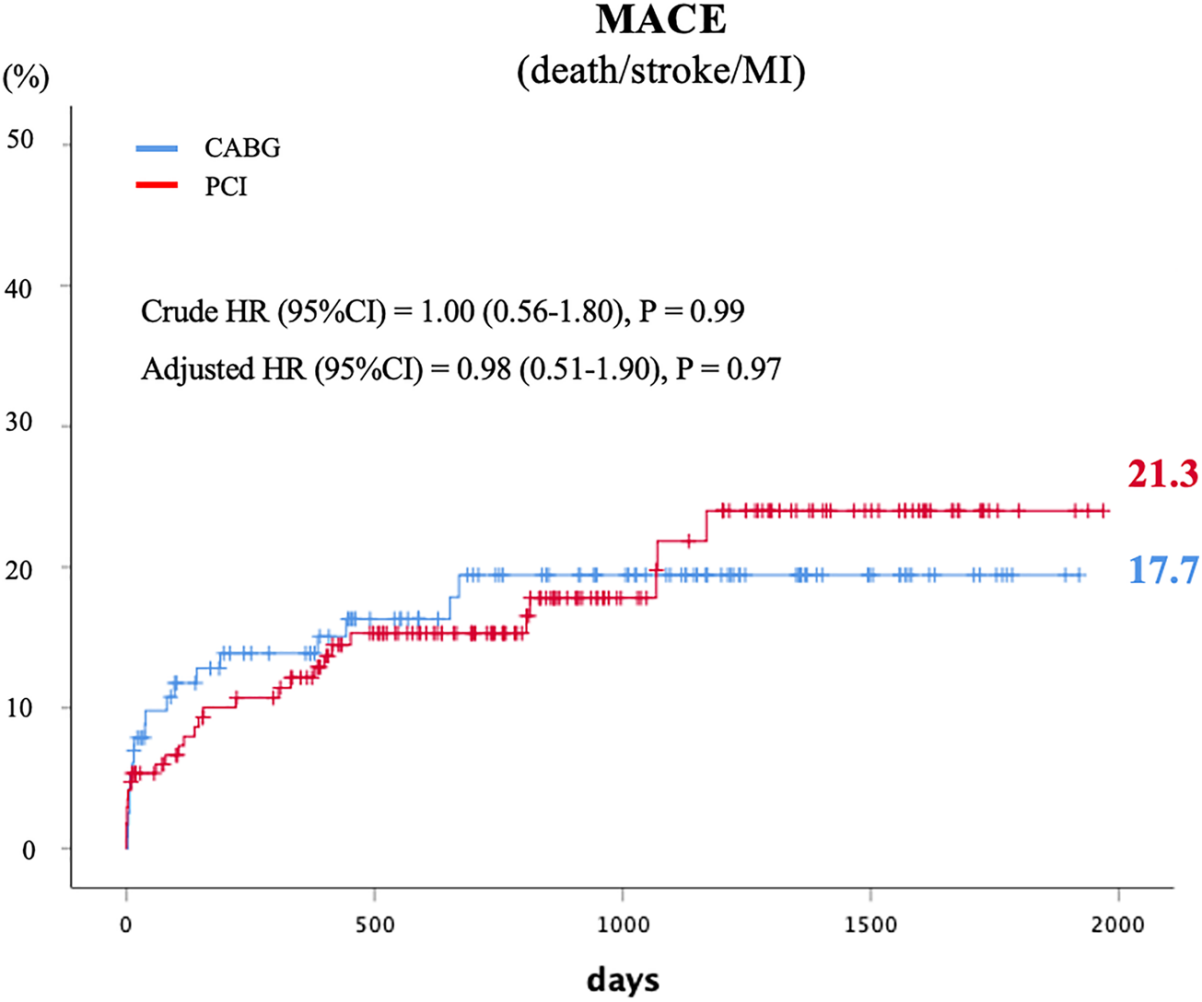
Cumulative MACE rates between PCI and CABG groups.

Table 3 presents clinical outcomes. After an average follow-up of 26.9 ± 17.7 months, the primary outcome occurred in 16.2% of PCI patients and 16.0% of CABG patients, a statistically nonsignificant difference. The hazard ratio (HR) (PCI vs. CABG) was 1.00 (95% confidence interval [CI], 0.56–1.80, *p* = 0.99) (Fig. 2). After adjusting for covariates (age, gender, diabetes, hypertension, chronic kidney disease, end-stage renal disease, known heart failure, prior MI), cumulative hazard rates of the primary outcome remained similar in both treatments, with an adjusted HR of 0.98 (95% CI, 0.51 to 1.90, p=0.97). Regarding the individual components of the primary outcome and other clinical endpoints, all-cause mortality (11.6% in PCI vs. 11.8% in CABG, p=0.93), stroke (3.5% in PCI vs. 5.0% in CABG, p=0.51), and cardiac death rates (6.9% in PCI vs. 6.7% in CABG, p=0.95) did not differ significantly. However, the MI rates (4.6% in PCI vs. 0.8% in CABG, p<0.05) and repeat revascularization rates (26% in PCI group vs. 5.9% in CABG, *p* < 0.001) were significantly higher in the PCI group.

**Table 3:** Clinical outcomes.

	PCI (n=173)	CABG (n=119)	Hazard ratio (95%CI) (PCI to CABG)	*P* value	Adjusted hazard ratio (95% CI) (PCI to CABG) ^+^	*P* value
Primary endpoint*	28 (16.2%)	19 (16.0%)	1.00 (0.56–1.79)	0.992	0.98 (0.51–1.90)	0.968
All-cause death	20 (11.6%)	14 (11.8%)	0.97 (0.49, 1.92)	0.929	0.78 (0.36–1.68)	0.539
Stroke	6 (3.5%)	6 (5.0%)	0.68 (0.22, 2.12)	0.512	0.66 (0.19–2.26)	0.512
MI	8 (4.6%)	1 (0.8%)	5.58 (0.69, 44.71)	0.047	25.22 (2.36–268.57)	0.007
Any revascularization	45 (26%)	7 (5.9%)	5.05 (2.27, 13.01)	<0.001	5.66 (2.46–13.01)	0.001
Cardiac death	12 (6.9%)	8 (6.7%)	1.02 (0.42, 2.51)	0.950	1.22 (0.47–3.17)	0.679

^*^ A composite of all-cause death, stroke, or MI.

^+^ Adjusted for age, gender, diabetes, hypertension, CKD, ESRD, known heart failure, prior MI. CABG: coronary artery bypass grafting; MI: myocardial infarction; PCI: percutaneous coronary intervention.

Table 4 shows the subgroup analysis. The event rate of the primary outcome did not significantly differ between PCI and CABG groups, irrespective of SS I and SS II classifications. The treatment effect of PCI versus CABG was consistent across all subgroups.

**Table 4:** Comparison of MACE between PCI and CABG in different subgroup.

	PCI (n=173)	CABG (n=119)	adjusted hazard ratio* (95% CI) (PCI to CABG)	*P* value	*P* for interaction
SS I					0.431
Low (< 22)	9/38 (23.7%)	3/24 (12.5%)	1.93 (0.52–8.14)	0.323	
Intermediate (23~32)	6/60 (10%)	6/37 (16.2%)	0.57 (0.18–1.77)	0.335	
High (> 33)	13/75 (17.3%)	10/58 (17.2%)	1.00 (0.43–2.28)	1.000	
SS II					0.511
PCI	1/23 (4.3%)	1/9 (11.1%)	0.41 (0.02–6.69)	0.538	
Equipoise	17/117 (14.5%)	8/76 (10.5%)	1.34 (0.58–3.11)	0.490	
CABG	10/33 (30.3%)	10/34 (29.4%)	1.06 (0.44–2.55)	0.897	
Gender					0.303
Male	15/139 (10.8%)	10/89 (11.2%)	0.92 (0.41–2.06)	0.852	
Female	13/34 (38.2%)	9/30 (30.0%)	1.40 (0.59–3.28)	0.440	
Diabetic mellitus					0.186
Yes	21/86 (24.4%)	11/59 (18.6%)	1.40 (0.67–2.91)	0.362	
No	7/80 (8.0%)	8/52 (13.3%)	0.55 (0.20–1.53)	0.257	
CKD					0.145
Yes	18/63 (28.6%)	10/41 (24.4%)	1.17 (0.54–2.54)	0.687	
No	10/110 (9.1%)	9/78 (11.5%)	0.76 (0.31–1.89)	0.569	
ESRD					0.294
Yes	11/19 (57.9%)	7/21 (33.3%)	1.76 (0.67–4.60)	0.237	
No	17/135 (11.0%)	12/98 (12.2%)	0.90 (0.43–1.90)	0.797	
Known heart failure					0.122
Yes	11/45 (24.4%)	4/10 (40%)	0.44 (0.14–1.43)	0.175	
No	17/128 (13.3%)	15/109 (13.8%)	0.94 (0.47–1.88)	0.863	
Clinical presentation					0.838
CCS	12/105 (11.4%)	10/65 (15.4%)	0.71 (0.30–1.65)	0.434	
UA	3/28 (10.7%)	2/17 (11.8%)	0.90 (0.15–5.42)	0.914	
NSTEMI	10/33 (30.3%)	5/31 (16.1%)	2.14 (0.73–6.28)	0.164	
STEMI	3/7 (42.9%)	2/6 (33.3%)	1.86 (0.29–11.73)	0.508	

^*^Adjusted for age, gender, diabetes, hypertension, CKD, ESRD, known heart failure, prior MI

**Figure 3:**
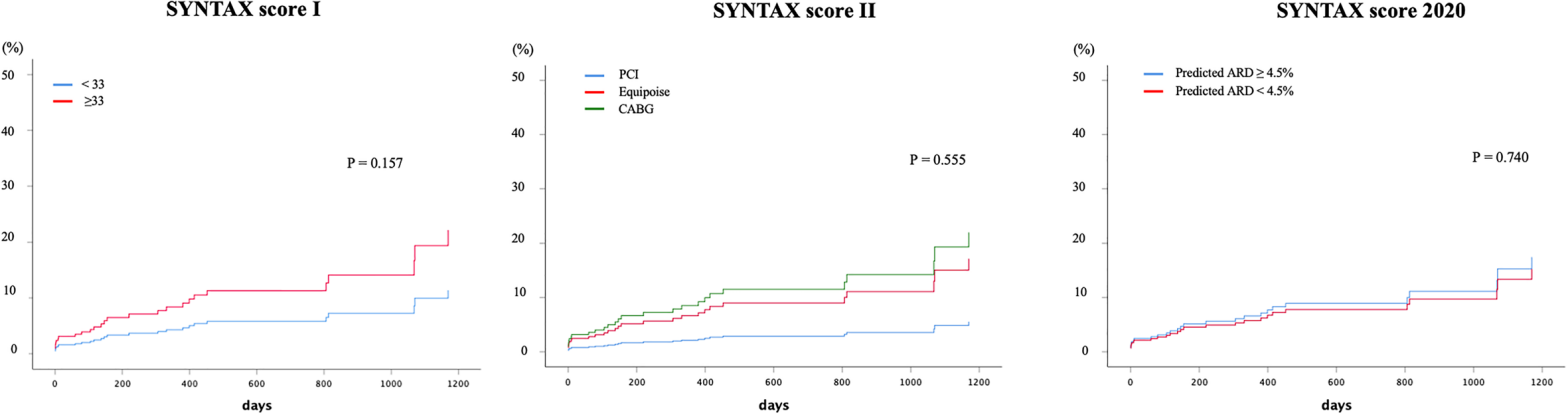
Cumulative MACE rates in the PCI group stratified by SS I, SS II, and SS II 2020.

Cox-regression analyses were further performed to investigate the association between MACE and SS I, SS II, and SS II 2020 subgroups in the PCI cohort respectively. Patients in the PCI group were stratified by SS I (<33 or ≥ 33), SS II recommendations (PCI, CABG, or equipoise) or SS II 2020 (ARD ≥ 4.5% or < 4.5%). Supplementary Table S2 shows the baseline characteristics of the PCI group divided by SS I (<33 or ≥ 33). Prognostic value of the SS I, SS II and SS II 2020 on the primary outcome was not relevant in our PCI cohort (Fig. 3 and Supplementary Table S3).

## Discussion

### Main findings

In this observational study, we identified that the incidence of all-cause death, stroke, or MI in patients with LMCAD undergoing either PCI or CABG was similar, even in those with a high SS I. Additionally, the rates of MI and repeat revascularization were significantly higher in the PCI group, corroborating the NOBLE trial’s results^16^.

The original SYNTAX trial suggested CABG as the preferable method for patients with LMCAD with high SS I^17,18^. Technological advancements, such as new drug coatings for DES and thinner metallic platforms^19^, have improved stent design, reducing the rate of in-stent restenosis and target lesion revascularization.

Moreover, the prevalent use of intravascular imaging, such as IVUS or OCT, has improved PCI outcomes^18,20,21^. The success of PCI for LMCAD relies on thorough pre-procedural planning, correct stent apposition, optimal stent expansion, and appropriate wire positioning during rewiring. Both IVUS^21–23^ and OCT^24,25^ can provide valuable information when performing PCI for LMCAD. Our cohort reported that 89.1% of patients undergoing PCI for LMCAD used intravascular imaging, higher than the 77.2% reported in the EXCEL trial^26^. Current guidelines recommend IVUS for LMCAD as a Class IIA intervention^27^.

These advancements in PCI techniques and technology have improved clinical outcomes, a conclusion supported by the results of the SYNTAX II study^28^. This progress also allows for a greater likelihood of complete revascularization, further improving clinical outcomes^29^. The complete revascularization rate of PCI in our study was 68.2%, which could explain the comparative outcome between PCI and CABG. In our study, most patients in the PCI group received DES (96.5%), all of which were second-generation or third-generation DES. This contemporary PCI approach for patients with LMCAD might explain the comparable short-term and mid-term outcomes between the PCI and CABG groups. As such, the applicability of only using the SS I to guide decision making on revascularization is questionable. It is noteworthy that real-world data show the advancements have made PCI an alternative choice for patients with LMCAD and high SS I^30^. Moreover, clinical factors such as diabetes or EuroSCORE were more relevant to outcomes instead of the SS I^31^.

Likewise, the mean SS I was 32.0 ± 11.6 in our study and 45.5% of patients had high SS I (SS I ≥ 33), supported PCI as a reasonable choice in high anatomical complexity cases who are ineligible for CABG.

Regarding the SS II 2020, the predicted 5-year MACE rates with PCI/CABG were both significantly higher in our PCI group than in the CABG group. It is noteworthy that patients receiving PCI in our study were older, with higher prevalence of heart failure and previous MI. This observation may reflect the fact that patients with a high risk may prefer PCI over CABG or even were not suitable for CABG after heart team evaluation.

Nevertheless, the PCI group had higher risks for MI and repeat revascularization, aligning with prior research^28,32–34^. Among the eight patients experiencing MI in the PCI group of our study, five incidents related to target lesion revascularization (TLR). In contrast, the CABG group reported a markedly lower MI rate. PCI primarily addresses flow-limiting lesions, but further events may occur due to either target lesions or non-target lesions. With CABG treatment, graft vessels usually bypass the entire disease vessels which might help avert future MI events^35^. Beside the diseased part of the target vessel, previous study suggested left internal mammary artery (LIMA) grafting was associated with lower risk of down-stream disease progression compared to PCI^36^. The EXCEL trial also confirmed significantly higher non-periprocedural MI rates in the PCI group compared with the CABG group at five years (6.8% vs. 3.5%)^3^.

In our cohort, most patients in the PCI group underwent repeat revascularization non-emergently, often for non-left main lesions. The risk of repeat revascularization and TLR did not vary between high and low to intermediate SS I group (supplementary Table S3). Furthermore, we suggest implementing standardized postoperative care after revascularization (PCI or CABG) in future studies due to potential disparities in postoperative care among surgeons and interventionalists.

The SS II and SS II 2020 incorporate clinical factors to predict long-term outcomes after revascularization. In our study, SS II recommendations did not significantly discriminate the risk of MACE in the PCI group. Similarly, a cut off value of ARD ≥ 4.5% or <4.5% calculated from the SS II 2020 did not associate with MACE. These observations may be influenced by a limited sample size without adequate statistical power. In addition, we used categorical variables rather than the absolute estimated mortality in this study, which limited the prognostic prediction. Patients with high estimated mortality in both PCI and CABG could be classified as equipoise in SS II and as ARD < 4.5% in SS II 2020, although their prognosis was expected to be poorer. The utility of SS II 2020 warrants further evaluation.

We acknowledge that our study, being retrospective and observational, may have inherent limitations, including potential selection bias and confounding factors. Periprocedural MI was not included due to debates over its definition after revascularization. Clinical events were obtained by reviewing medical records without formal adjudication. Bleeding events were not systematically collected and reported. The residual SYNTAX score was not provided in this study.

## Conclusion

In this single-center retrospective study, we observed no significant difference in the composite outcome of all-cause death, stroke, or MI between PCI and CABG in patients with LMCAD, irrespective of SS I. These results support the potential expansion of PCI indications in LMCAD management for whom are ineligible for CABG with complex coronary artery disease.

### Supplementary Information

Supplementary Information.

## Data Availability

The datasets used and analyzed during the current study available from the corresponding author on reasonable request.
