# Supplementary material for: Impacts of the SYNTAX score I, II and SYNTAX score II 2020 on left main revascularization
Source: Sci Rep. 2024 Jan 11;14:1073. doi: 10.1038/s41598-024-51192-7 (PMC10784526; doi:10.1038/s41598-024-51192-7)
Supplement: Supplementary file 1 — Supplementary Information. [file 41598_2024_51192_MOESM1_ESM.docx]

**Supplementary table S1. Procedural details of PCI group**

|  | **All**  (n=173) | **SS I 0-32**  (n=98) | **SS I ≥33**  (n=75) | **P value** |
| --- | --- | --- | --- | --- |
| Complete revascularization | 118 (68.2%) | 73 (74.5%) | 45 (60.0%) | 0.043 |
| Image guided PCI | 155 (89.1%) | 86 (87.8%) | 69 (92.0%) | 0.365 |
| IVUS | 144 (83.2%) | 80 (81.6%) | 64 (85.3%) | 0.518 |
| OCT | 12 (6.9%) | 7 (7.1%) | 5 (6.7%) | 0.903 |
| One-stent technique | 122 (70.5%) | 67 (68.4%) | 55 (74.7%) | 0.478 |
| Two-stent technique | 52 (30.1%) | 31 (31.6%) | 20 (26.7%) | 0.478 |
| T or TAP | 11 (6.4%) | 5 (5.1%) | 6 (8.0%) | 0.439 |
| Culotte | 30 (17.3%) | 20 (20.4%) | 10 (13.3%) | 0.223 |
| Mini crush | 9 (5.2%) | 6 (6.1%) | 3 (4.0%) | 0.533 |
| DK crush | 3 (1.7%) | 1 (1.0%) | 2 (2.7%) | 0.411 |
| KBT performed | 83 (51.2%) | 47 (54.0%) | 36 (48.0%) | 0.444 |
| POT performed | 152 (87.9%) | 85 (86.7%) | 67 (89.3%) | 0.604 |
| DES | 167 (96.5%) | 93 (94.9%) | 74 (98.7%) | 0.179 |

Data are n (%); DES: drug eluting stent; DK: double kissing; IVUS: intravascular ultrasound; OCT: optical coherence tomography; PCI: percutaneous coronary intervention; POT: proximal optimization technique; SS I: SYNTAX score I; TAP: T and protrusion.

|  | **SS I < 33**  (n=98) | **SS I** ≥**33**  (n=75) | ***P* value** |
| --- | --- | --- | --- |
| Mean age, years (SD) | 72.3 ± 12.6 | 71.6 ± 12.0 | 0.709 |
| Male | 71 (72.4%) | 67 (89.3%) | 0.007 |
| **Medical history** |  |  |  |
| Diabetes mellitus | 51 (52.0%) | 35 (46.7%) | 0.540 |
| Hypertension | 75 (76.5%) | 59 (78.7%) | 0.855 |
| Known HF | 25 (25.5%) | 20 (26.7%) | 0.863 |
| COPD | 8 (8.2%) | 5 (6.7%) | 0.779 |
| Chronic kidney disease | 38 (38.8%) | 25 (33.3%) | 0.525 |
| End staged renal disease | 13 (13.3%) | 6 (8.0%) | 0.332 |
| Previous stroke | 8 (8.2%) | 14 (18.7%) | 0.064 |
| Previous myocardial infarction | 17 (17.3%) | 13 (17.3%) | 1.000 |
| Previous PCI | 29 (39.8%) | 21 (28.0%) | 0.111 |
| PAD | 11 (11.2%) | 6 (8.0%) | 0.609 |
| **Clinical presentation** |  |  | 0.759 |
| Stable coronary artery disease | 61 (62.2%) | 44 (58.7%) |  |
| Unstable angina | 17 (17.3%) | 11 (14.7%) |  |
| NSTEMI | 17 (17.3%) | 16 (21.3%) |  |
| STEMI | 3 (3.1%) | 4 (5.3%) |  |
| LVEF (%) | 53.8 ±13.0 | 48.7 ±14.5 | 0.019 |

**Supplementary table S2. Baseline characteristics of the PCI group**

Data are mean ± SD or n (%); AF: atrial fibrillation; CABG: coronary artery bypass grafting; COPD: chronic obstructive pulmonary disease; HF: heart failure; NSTEMI: non-ST elevation myocardial infarction; PAD: peripheral artery disease; PCI: percutaneous coronary intervention; STEMI: ST elevation myocardial infarction.

**Supplement Table S3. Clinical outcomes stratified by SS I**

|  | **SS I <33**  (n=98) | **SS I ≥33**  (n=75) | **adjusted hazard ratio^++^**  **(95% CI)** | **P value** |
| --- | --- | --- | --- | --- |
| MACE^*^ | 15 (15.3%) | 13 (17.3%) | 1.85 (0.79-4.34) | 0.157 |
| All-cause death | 12 (12.2%) | 8 (10.7%) | 1.46 (0.53-4.04) | 0.470 |
| Stroke | 4 (4.1%) | 2 (2.7%) | 0.51 (0.09-3.01) | 0.454 |
| MI | 4 (4.1%) | 4 (5.3%) | 1.05 (0.18-6.21) | 0.957 |
| Cardiac death | 8 (8.2%) | 4 (5.3%) | 1.39 (0.34-5.70) | 0.647 |
| TLR^+^ | 13 (13.3%) | 8 (10.7%) | 0.73 (0.29-1.85) | 0.511 |
| Any revascularization | 19 (19.4%) | 24 (32%) | 1.62 (0.88-2.99) | 0.125 |

Data are n (%); * A composite of all-cause death, stroke, or MI. ^+^ Target lesion is referred to left main coronary artery. ^++^ Adjusted for age, gender, diabetes, hypertension, CKD, ESRD, known heart failure, prior MI, and prior stroke.

**Supplementary table S4 Clinical outcomes stratified by acute coronary syndromes**

| **Non-ACS (stable coronary artery disease, unstable angina)** | | | |  | |
| --- | --- | --- | --- | --- | --- |
|  | **PCI**  **(n=133)** | **CABG**  **(n=82)** | **Hazard ratio (95%CI)**  **(PCI to CABG)** | | ***P* value** |
| Primary endpoint* | 15 (11.3%) | 12 (14.6%) | 0.748 (0.350, 1.599) | | 0.457 |
| All-cause death | 8 (6.0%) | 8 (9.8%) | 0.603 (0.314, 1013)) | | 0.314 |
| Stroke | 5 (3.8%) | 5 (6.1%) | 0.592 (0.171, 2.048) | | 0.411 |
| MI | 4 (3.0%) | 1 (1.2%) | 2.458 (0.274, 22.071)) | | 0.384 |
| Any revascularization | 36 (27.1%) | 5 (6.1%) | 4.692 (1.839, 11.969) | | 0.001 |
| Cardiac death | 6 (4.5%) | 5 (6.1%) | 0.723 (0.221, 2.370) | | 0.593 |
| **ACS (Non-STEMI, STEMI)** | | | | | |
|  | **PCI**  **(n=40)** | **CABG**  **(n=37)** | **Hazard ratio (95%CI)**  **(PCI to CABG)** | | ***P* value** |
| Primary endpoint* | 13 (16.9%) | 7 (9.1%) | 2.031 (0.809, 5.101) | | *0.132* |
| All-cause death | 12 (15.6%) | 6 (7.8%) | 2.132 (0.799, 5.690) | | *0.131* |
| Stroke | 1 (2.5%) | 1 (2.7%) | 1.350 (0.083, 21.594) | | *0.832* |
| MI | 4 (10%) | 0 (0%) | 68.316(0.022, 213051) | | *0.018* |
| Any revascularization | 9 (22.5%) | 2 (5.4%) | 5.203 (1.119, 24.191) | | *0.035* |
| Cardiac death | 6 (7.8%) | 3 (3.9%) | 2.169 (0.540, 8.706) | | *0.275* |

* A composite of all-cause death, stroke, or MI.

ACS: acute coronary syndrome; CABG: coronary artery bypass grafting; MI: myocardial infarction; PCI: percutaneous coronary intervention; STEMI: ST-elevation myocardial infarction


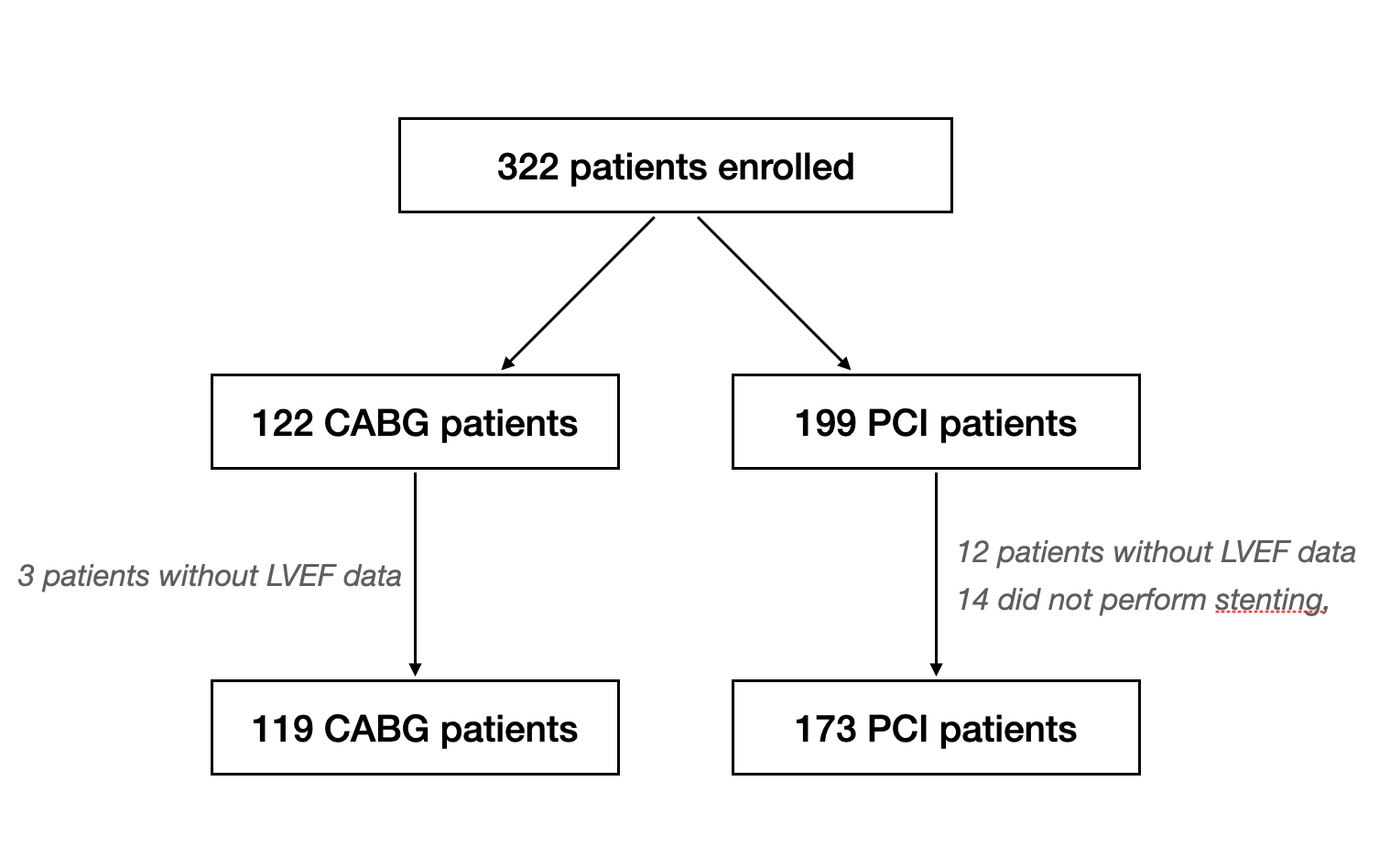


*Figure S1. Flow chart of data collection.*
